# From Secular Isolation to Current Globalisation: Preserving the Ethnobotanical Knowledge in Eivissa/Ibiza (Balearic Islands, Spain)

**DOI:** 10.3390/plants14060890

**Published:** 2025-03-12

**Authors:** Raquel González, Teresa Garnatje, Joan Vallès

**Affiliations:** 1 Laboratori de Botànica—Unitat Associada CSIC, Facultat de Farmàcia i Ciències de l’Alimentació—IRBio, Universitat de Barcelona, Av. Joan XXIII 27–31, 08028 Barcelona, Catalonia, Spain; joanvalles@ub.edu; 2 Institut Botànic de Barcelona (IBB), CSIC-CMCNB Passeig del Migdia s.n., Parc de Montjuïc, 08038 Barcelona, Catalonia, Spain; tgarnatje@ibb.csic.es; 3 Jardí Botànic Marimurtra-Fundació Carl Faust, Passeig Carl Faust, 9, 17300 Blanes, Catalonia, Spain; 4 Institut d’Estudis Catalans (IEC), Carrer del Carme, 47, 08001 Barcelona, Catalonia, Spain

**Keywords:** Balearic Islands, Eivissa, ethnobotany, Ibiza, traditional knowledge, medicinal plants, landraces

## Abstract

Eivissa/Ibiza, as per its names in its two official languages, Catalan and Spanish, is the third of the Balearic Islands in terms of extension and the second concerning population. It is also a well-known holiday destination in Europe. Numerous ethnobotanical prospections have been performed in the Balearic Islands, but to date, Ibiza lacks a monographic study on traditional knowledge related to plant biodiversity. In this paper, we present the results of the ethnobotanical investigation carried out in Ibiza from 2016 to 2023. A total amount of 95 interviews were conducted with 101 informants born between 1916 and 1983, with semi-structured interviews, participant observation and plant collection, identification and deposit in a public herbarium as basic methods. The total ethnoflora of the island is 254 taxa belonging to 71 botanical families. The most cited families are *Solanaceae* (1030 URs, 13.50%), followed by *Fabaceae* (770 URs, 10.09%), *Lamiaceae* (646 URs, 8.47%) and *Rutaceae* (578 URs, 7.57%). The most cited species are *Vitis vinifera*, *Capsicum annuum*, *Solanum lycopersicum*, *Solanum tuberosum* and *Citrus sinensis*. This study reveals that the local population still retains significant ethnobotanical knowledge. Further research in similar territories could help determine whether this pattern is consistent elsewhere.

## 1. Introduction

Ethnobotany is a science that studies humans’ relationships with plants around them, gathering traditional knowledge about the use of those plants and information associated with them. Ethnobotany, a term coined and first defined by Harshberger [1], is placed in the interface between botany and ethnography, using both human and social science methodologies ([2,3] and references therein). As stated by Gispert et al. [4], ethnobotany is a frontier discipline rescuing and updating plants’ history in human societies through time and space.

The island studied is known as Ibiza in Spanish and Eivissa in Catalan; hereafter, we will use Ibiza in the text, since it is more extensively recorded in the international literature, whereas in the title and the abstract, both names are reflected.

**Figure 1:**
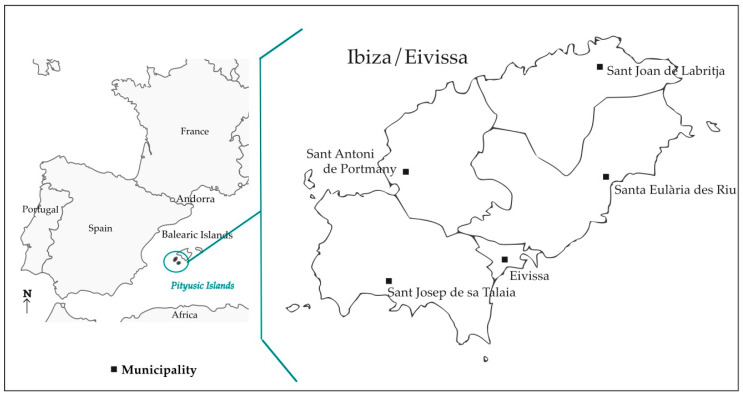
Location of the studied area.

Located to the east of the Iberian Peninsula (Figure 1), Ibiza was founded by the Punics, who, among other plants and products, brought *Vitis vinifera* there in the 7th century BC, creating structures purposely for cultivating grapevines and producing wine [5]. From then on, Ibiza has been frequently visited by various civilisations and has participated in horticultural commerce, including grapes and wine [6]. Nevertheless, even if not at all absolute, a certain degree of isolation (reinforced, in the etymological sense, by the fact that Ibiza is an island) has resulted in a particular linguistic and, in general, cultural heritage, including knowledge and management of plants and plant-derived products [7,8,9]. Both aspects, relationships and isolation, were probably significant reasons why Ibiza was classified as a UNESCO World Heritage Site in 1999 and has been part of the Cultural Landscape Alliance since 2018.

Agriculture has been the basis of Ibiza’s economy from the earliest times, but the situation changed dramatically in the second half of the 20th century, very quickly transforming from a rural subsistence economy to a service sector-based economy, with tourism as practically the only driving force [10,11,12]. Particularly, between 1965 and 1975, the active agrarian population was reduced from 46 to 18%, and the cultivated surface decreased by 25% from 1962 to 1980 [10]. This situation continued, and in 2006, the percentage of the active population working in agriculture was 1.4% [13], whereas nowadays, it is 1.01% [14]. Despite the decrease in cultivated land due to the rise in tourism and the urbanisation of flatter areas, today, ca. 4213 ha are devoted to plant cultivation on the island, with almond and other dry fruits, vines, wheat, forage and sweet fruits in significant quantities [15].

The Pityusic Islands, the name by which Ibiza and Formentera are collectively known, are part of the Balearic Islands and are also included in the Catalan linguistic area (CLA), i.e., the area where Catalan language is spoken, in the occidental part of the Mediterranean region. This region is one of the best-prospected zones in Europe from an ethnobotanical point of view ([3] and references therein). Different works have been carried out in these territories from various countries, including Spain (Catalonia, Valencian Community, Balearic Islands, the eastern strip of Aragon, and Carxe, a small part of Murcia community), France (Northern Catalonia or Eastern Pyrenees department), Andorra (the whole country), and Italy (the city of l’Alguer, on Sardinia island).

The entire Balearic archipelago has been significantly and quite extensively studied from an ethnobotanical perspective ([16] and references therein). However, most research has focused either on the whole archipelago (with a predominance of the largest island, Mallorca) or on the Gymnesian islands, i.e., Mallorca and Menorca [17,18,19,20,21,22,23,24,25,26,27,28]. In contrast, studies documenting traditional plant knowledge specific to the Pityusic Islands are relatively scarce despite significant botanical research being reported for this smaller subarchipelago [29]. For Formentera, the smallest of the islands, ethnobotanical data are present in Guerau d’Arellano and Torres [30,31,32,33,34,35,36], Torres [8], Carrió et al. [23], Gras et al. [27], and Torres et al. [37].

Ethnobotanical research on Ibiza began in the early 20th century with studies on *Corylus avellana* and agricultural plants [38,39,40]. Key works include documentation of folk plant names and their uses by Guerau d’Arellano and Torres [30,41], updated by Torres et al. [37], a field guide to Pityusic flora [42], and notes on local plant names [31,32,33,34,35]. Guerau d’Arellano studied taxa management [29,43], while Planells [44] and Gómez [45] examined medicinal plants in folk medicine. A comprehensive list of the botanical literature on the Pytiusic Islands until the mid-1980s can be found in Guerau d’Arellano [29].

A very interesting contribution to the knowledge of the ethnobotanical heritage in Ibiza is the anthropological study carried out by Torres [8,46], addressing beliefs, costumes and festivals, as well as plants’ roles in shepherding and traditional rituals. Additionally, research on agriculture and cuisine, as well as other aspects of plant uses, such as fibre, also contributes to ethnobotanical insights [10,47,48,49,50,51,52,53,54,55,56,57]. Finally, popular culture, such as sayings, some of which deal with plants [58], also brings ethnobotanical data. The study of cultivated plant landraces in the Balearic Islands [59] encompasses Ibiza; however, a comprehensive ethnofloristic survey, such as Mayans’ [60] work on Formentera, has yet to be undertaken.

Due to the lack of a robust ethnobotanical prospection in Ibiza and the urgency of compiling its ethnoflora, the objective of this work is to contribute to the collection of traditional knowledge about plants, their common names, and their uses on the island of Ibiza for subsequent study and preservation, as well as to compare Ibiza’s ethnoflora with those reported in other areas, particularly the remaining Balearic, Iberian and western Mediterranean territories.

Ibiza’s distinctive relationships, as mentioned above, and its relative isolation were likely significant factors in its designation as a UNESCO World Heritage Site in 1999 and its inclusion in the Cultural Landscape Alliance in 2018. Despite being included in studies on cultivated plant landraces in the Balearic Islands [59], Ibiza has yet to undergo a comprehensive ethnofloristic survey akin to Mayans’ [60] work in Formentera. This gap in detailed ethnobotanical research underscores the urgent need to document Ibiza’s ethnoflora. Therefore, this study aims to collect traditional knowledge about plants, including their common names and uses on the island. It also seeks to support ongoing research and preservation efforts while facilitating comparisons of Ibiza’s ethnoflora with those of other regions, particularly the other Balearic Islands, the Iberian Peninsula, and the western Mediterranean.

## 2. Results and Discussion

### 2.1. Characteristics of the Informants

A total of 95 interviews were conducted with 101 informants born between 1916 and 1983. Table 1 shows the number of interviews conducted and individuals interviewed per municipality. Of the total number of interviewees, 60 (59.41%) were male, while the remaining 41 (40.59%) were female. In the year 2022, the population of Ibiza was 154,210 inhabitants, with 78,524 being men (50.92%) and 75,686 women (49.08%) [14], so our prospection was only a little biased toward men. The percentages and distribution are practically identical in the ethnobotanical work carried out on another Balearic island, Mallorca (60% men, 40% women [26]), whereas on the smaller Pityusic Island of Formentera, 79% of interviewees were women, and only 21% were men [60]. In other areas of the CLA, a predominance of women was also observed in most areas of Catalonia [61,62,63,64], whereas in one case [65], the percentages (59.41% men, 40.59% women) were almost identical to those in the present paper, with a predominance of men. In Iberian Spanish-speaking territories, a prevalence of men—in some cases very significant—has also been observed [66,67,68], although not in all cases [69]. Finally, in an Iberian Portuguese-speaking area, a big predominance of women is observed [70], and, to a lesser extent, this also happens in an Iberian area with Spanish- and Basque-speaking populations [71]. With this panorama, we can state, just as an intuitive and qualitative observation, that in Iberian Spanish-speaking territories, there is a predominance of men as informants, whereas the situation is reversed in Iberian areas with the presence of another language. One of the points that can explain the predominance of men as informants in some areas [72] is the dominant role of the man in the family, which even in some cases can overshadow the woman during the interview or before it takes place. Also, the scarce presence of women in some public places (such as bars, where interviews are rarely carried out) in rural areas could reinforce this idea. The higher proportion of women as informants, who are the majority in the CLA, has been considered logical [73] since, in general, they take care of the house, the kitchen, the home garden and similar activities closely linked to plants and their traditional management. Finally, a bias in informants’ selection, even if, in almost all cases, a snowball approach is followed, could also influence the proportions. In our case, the discrepancy from most of the research in the CLA is probably due to the relevance of agricultural ethnobotany, including landraces, as a focus of our prospection (although it has globally been a general one), the knowledge of which is almost uniquely in the hands of men.

Out of the 101 informants, 67 (66.36%) were already retired at the moment of the interview. The main professions of the interviewees were linked to the agropastoral and, in general, rural sectors, such as farmers, town housewives and shopkeepers (especially in shops dealing with food), although there was a significant representation of service sector workers, most of whom were engaged in tourism, reflecting the above-mentioned social changes experienced in the socioeconomy of the island.

### 2.2. Taxa, Botanical Families, and Use Reports

In this study, 254 taxa (237 species belonging to 192 genera), including 136 belonging to wild plants and 118 to cultivated, have been reported, of which six have been identified only at the genus level, and 27 exhibit infraspecific categories. The complete list of taxa and their uses can be found in Supplementary Materials

**Figure 2:**
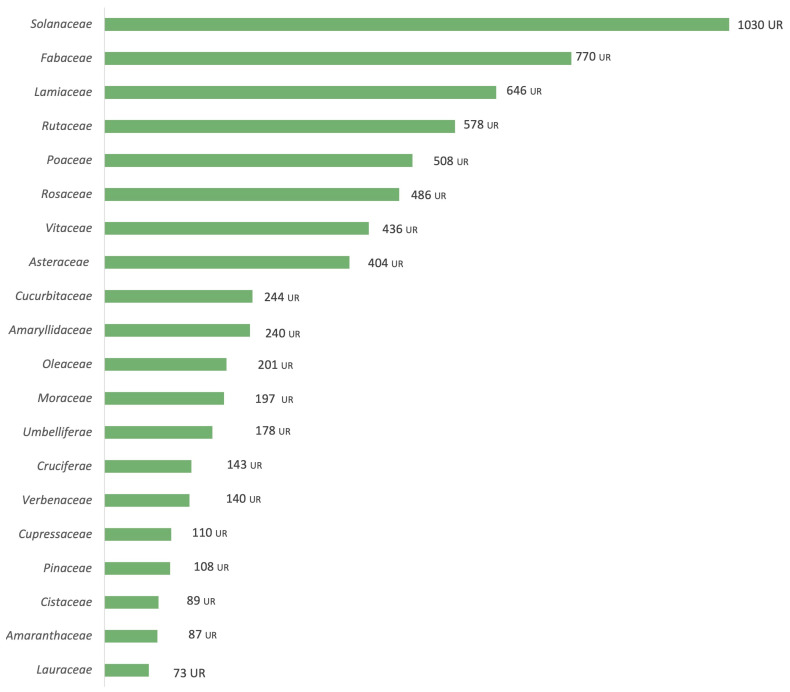
The most cited botanical families.

These taxa belong to 71 botanical families, the most cited (Figure 2) being *Solanaceae* (1030 URs, 13.50%), followed by *Fabaceae* (770 URs, 10.09%), *Lamiaceae* (646 URs, 8.46%), *Rutaceae* (578 URs, 7.58%), *Poaceae* (508 URs, 6.66%), *Rosaceae* (486 URs, 6.37%), *Vitaceae* (436 URs, 5.71%) and *Asteraceae* (404 URs, 5.29%). The prevalence of these families in the reported data can be attributed to two primary factors. Firstly, these families boast a diverse array of species within their ranks, contributing to their prominence in biodiversity reports. Secondly, their widespread distribution and dominance in the Mediterranean basin further underscore their significance in the collected data. In fact, *Asteraceae* and *Lamiaceae* are almost always among the most cited in similar works in Mediterranean territories [74,75].

Of particular interest is the noteworthy inclusion of the *Solanaceae* family, marking a distinctive finding. This can be elucidated by Ibiza’s enduring agricultural landscape, where this family holds a robust presence. It is essential to acknowledge the potential existence of a slight bias in the reported findings, as this study, although including a general ethnobotanical prospection, has delved extensively into cultivated species, potentially influencing the prevalence of certain families in the results. The family *Solanaceae* contains several genera of food and agricultural interest, among which *Capsicum* and *Solanum* are relevant for the number of uses and races. The families *Fabaceae* and *Poaceae* also include many cultivated plants, with the latter being also important in terms of wild plants in the biogeographic region the studied area belongs to. *Poaceae* is far from one of the top families in the Catalan and Iberian ethnobotany, but not absolutely, since it occupies the fifth place in the family ranking for a Catalan inland semiarid region [65] and second in another territory heavily influenced by agricultural activity [76].

The informants have provided 7628 URs, of which 483 URs are associated with 76 taxa (12 infraspecific categories) for medicinal uses, 2569 URs (158 taxa, 12 infraspecific categories) for food uses, and 507 URs (110 taxa, 12 infraspecific categories) for other purposes. It is worth mentioning that some taxa are common to two or three of these categories.

The ethnobotanicity index [77] for only wild (including naturalised) plants is 12.01%, roughly indicating that around one-eighth of the Ibizan flora is constituted by plants having almost one kind of traditional use. This index was calculated based on the estimated number of wild taxa, as described in the Section 3, and considering wild plants that have been reported as such on the island in the literature cited in the Section 1 and Section 3, even though in some cases (such as, among others, *Artemisia arborescens* L., *Ceratonia siliqua* L. or *Santolina chamaecyparissus* L.), at least some (if not all) of the material used by the informants may have come from cultivation. The present value is among the lowest in the CLA ([78,79] and references therein) but is not far from the one obtained in a region of another Balearic island, Mallorca (15.5%) [24]. In the present study, the number of cultivated taxa known and used by the informants (108) is almost as significant as that of wild plant taxa (131).

The general informant consensus factor [80] for all cited plants and their uses is 0.97. It reaches the top in the ranking of this parameter in the CLA (from 0.71 to 0.96 [76] and references therein), indicating a very high consistency in plant uses in the considered territory and, so, showing the robustness of the obtained dataset. A total of 161 plant taxa (around two-thirds of the total number) have been cited by at least three independent informants, reaching the quality criterion of Le Grand and Wondergem [81] and Johns et al. [82], which makes them candidates for further studies and large-scale uses. Nevertheless, the plants cited only by one or two informants should not be neglected since, on the one hand, they are also interesting from a biocultural viewpoint, and, on the other hand, their relatively low level of quotation may be due to the acculturation and the consequent break of the transmission chain for traditional knowledge in industrialised areas.

### 2.3. Medicinal Uses

**Figure 3:**
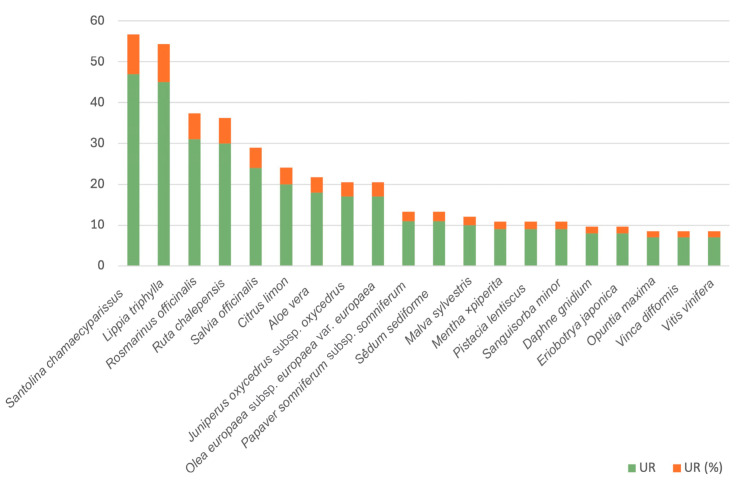
The most cited taxa with medicinal uses.

The number of medicinal use reports is 483, corresponding to 75 species (12 taxa at the infraspecific level), 47 wild plants and 28 cultivated plants, including 67 genera and 37 families. Figure 3 shows the 20 most cited taxa with medicinal uses. All taxa, uses and numbers of the URs are listed in Table S1 of Supplementary Materials. The medicinal importance index (quotient of the total medicinal use reports and the number of medicinal taxa) is 6.36. The informant consensus factor for medicinal uses is 0.84, similar to that presented in other areas of the CLA ([65,79] and references therein) and higher than the one reported for other Mediterranean (0.72 [83]; 0.72 [84]) or Mesoamerican (0.75 [85]; 0.79 [86]) territories. This confirms the consistency of the dataset provided by the informants, which has already been commented regarding the general informant consensus factor.

The most frequently referenced species is *Santolina chamaecyparissus*, accounting for 47 URs (9.73%). Belonging to the *Asteraceae* family, this plant’s inflorescences find common use, with a prevalent practice of utilising them in their dried form for crafting infusions aimed at addressing various digestive system disorders. These infusions demonstrate efficacy as intestinal anti-inflammatories, antiemetics, and general digestive aids. Additionally, *Santolina chamaecyparissus* has earned recognition for its potential as an anticatarrhal agent, addressing associated symptoms, such as antipyretic and anticephalalgic properties. Moreover, it serves as a tranquilliser. To a lesser extent, this versatile species has also been acknowledged for its use as an eye antiseptic. Infusions are employed for eye baths, demonstrating effectiveness in treating conditions like conjunctivitis.

The second most referenced species is *Lippia triphylla* (L’Hér.) O.Kuntze (*Verbenaceae*), registering 45 URs (9.32%). These plants’ leaves and flowering aerial parts are employed for purposes similar to those of the previously mentioned species. Notably, in addition to the established uses, *Lippia triphylla* extends its therapeutic applications to include antidiarrheal properties. Furthermore, it has collected citations for its potential cardiotonic effects, adding to its diverse range of recognised uses.

*Rosmarinus officinalis* L. (*Lamiaceae*) is in the third position, with 31 URs, representing 6.42% of references. The leaves and, broadly, the aerial parts of this plant have earned citations for their analgesic, antiseptic, anti-inflammatory, and wound-healing properties, making them a common recommendation for addressing trauma. In the diverse spectrum of pharmaceutical applications, a noteworthy preparation method stands out, referred to by informants as “rosemary oil” in translation, despite its lack of actual oil content. In this unique formulation, rosemary leaves are placed in a bottle and left exposed to the sun and air (“sol i serena” in Catalan). The resulting liquid from this natural process is then applied to injuries and traumas, showcasing a distinctive and traditional method of utilising rosemary for therapeutic purposes.

The leaves and aerial components of *Ruta chalepensis* subsp. *chalepensis* L. (*Rutaceae*) have garnered significant attention, with 30 URs accounting for 6.21% of references. Notably, they have been widely recognised for their antidiarrheal and anti-inflammatory properties, particularly concerning intestinal health. Additionally, this plant has been acknowledged for its anthelmintic qualities, contributing to its multifaceted therapeutic reputation. However, the most noteworthy and simultaneously discreet applications of this taxon pertain to women’s health, typically administered in the form of an infusion. It has been historically employed to alleviate menstrual pain and, notably, recognised for its abortive properties.

The leaves of *Salvia officinalis* L. (*Lamiaceae*) (24 URs, 4.97%), securing the fifth position among the most cited species for medicinal applications in the form of infusions, are used as an anti-inflammatory and oral antiseptic. However, their standout attribute lies in their capacity to act as an antihypertensive agent, being particularly esteemed for their role in lowering blood pressure.

The most cited families are *Lamiaceae* (75 URs, 15.53%), *Asteraceae* (68 URs, 14.08%), *Rutaceae* (57 URs, 11.80%), and *Verbenaceae* (45 URs, 9.32%), sharing the fifth place, with the same number of use reports as *Oleaceae* and *Rosaceae* (22 URs, 4.55%). As stated in the general results, the first two families are usually among the most cited in the Mediterranean territories. Here, we do not find the family *Solanaceae* as predominating, as was the case in the general results, proving that food use was responsible for its first place in the ranking. The high number of citations for *Rutaceae* is mainly due to the importance of *Ruta chalepensis* subsp. *chalepensis*, which, as mentioned above, is cited for various ailments (as an anti-inflammatory, an anthelmintic, for disorders of the digestive system and as an abortive, among others), but also due to the use of citrus fruits (mainly *Citrus limon* and *C. sinensis*), especially in the form of fruit juice, with anti-inflammatory, antiseptic, and anticatarrhal properties. The use reports of the *Oleaceae* are mainly due to the use of the oil extracted from the fruit of the olive tree (*Olea europaea* L. subsp. *europaea* var. *europaea*), often used as an excipient or as a vehicle in various preparations or pharmaceutical forms. The *Rosaceae* family includes three species with great relevance in traditional Ibizan medicine, *Eriobotrya japonica* (Thunb.) Lindl., *Rubus ulmifolius* Schott and *Sanguisorba minor* Scop.

**Figure 4:**
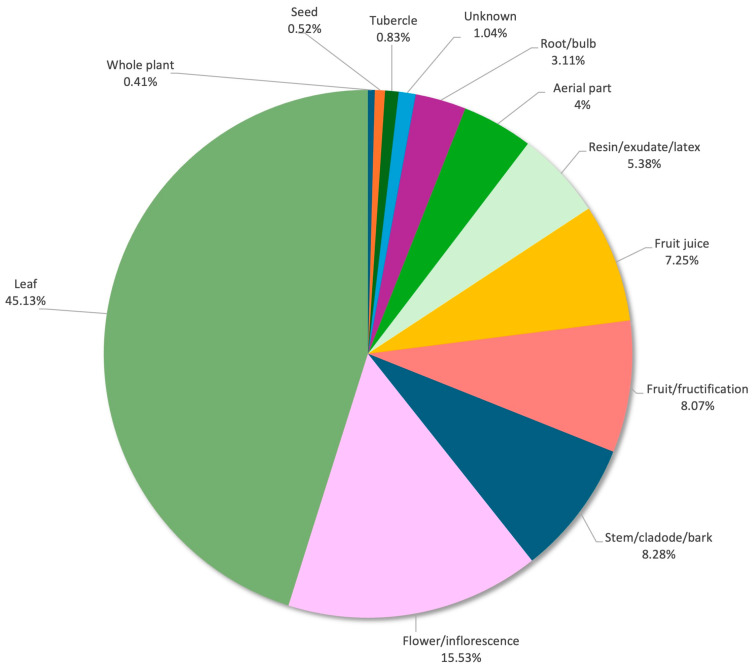
The most cited parts of plants with medicinal uses.

The parts of a plant (Figure 4) with the most medicinal uses are the leaves (216 URs, 44.72%), followed by the flowers and inflorescences (73 URs, 15.11%), the fruits and fructification (40 URs, 8.28%) and the stem, cladodes and bark (40 URs, 8.28%). All these are aerial parts, which are among the more visible and clearly identifiable parts of plants, making people more confident to collect and use them [25].

The most cited disorders are those of the digestive system (141 URs, 29.19%). This comprehensive category encompasses various conditions, such as oral, gastric, and intestinal inflammation, as well as antiemetic, digestive, antidiarrheal, antiodontalgic, and oral antiseptic needs. Additionally, these remedies extend their efficacy to address issues like flatulence and provide relief from jaundice.

In second place, disorders of the respiratory system are cited (65 URs, 13.46%), which primarily include uses against bronchitis, anticatarrhals, pharyngeal anti-inflammatories, antitussives, and expectorants. They are followed by infections and infestations (41 URs, 8.49%), which include the fight against the causative agent (antibacterial and anthelmintic), but they are also used to alleviate the symptoms, such as antipyretics. Disorders of the skin or subcutaneous tissue (38 URs, 7.87%) mainly include external antiseptics and wound healing, but also a few URs for treating psoriasis. Disorders of the cardiovascular system (35 URs, 7.25%) include almost exclusively the use of antihypertensives and cardiotonics, the latter residually.

The most cited form of application is internal (290 URs, 60.04%). The external form represents 174 URs (36.02%), and the remaining 3.94% is unknown. Infusion (221 URs, 45.76%) and direct use without pharmaceutical form (105 URs, 21.74%) represent 67.49% of the total number of reports. The rest of the pharmaceutical forms have much lower percentages (Table 2).

Despite the fact that the medicinal (veterinary) uses for animals have been very scarcely cited (31 URs, 6.42%), the uses of external antiseptic, antidiarrheal, insect repellent and birth coadjuvants should be highlighted.

### 2.4. Food Uses

A total number of 151 species (158 taxa, 17 of which correspond to infraspecific categories), including 101 cultivated plants and 50 wild plants, included in 121 genera and 49 families, have been collected for food uses, encompassing edible plants, those used as culinary aromatics, seasonings, preservatives or similar, and those employed to prepare various types of drinks. Species with such uses, which accumulate a number of 2569 URs, are detailed in Table S2 in Supplementary Materials. Table 3 displays the top 20 most cited species. The food importance index (the quotient of the total food use reports and the number of food taxa), an adaptation of the medicinal importance index for the food category [25], is 16.25, which is much higher than those corresponding to medicinal and other use categories, showing the strength of food uses on the island and also accounting for the relevance given to ethnoagronomic issues in the present work. The informant consensus factor for food uses is 0.94, which is very high, showing, again, the consistency and robustness of the dataset, particularly in food uses, which are more prominent in this work.

**Figure 5:**
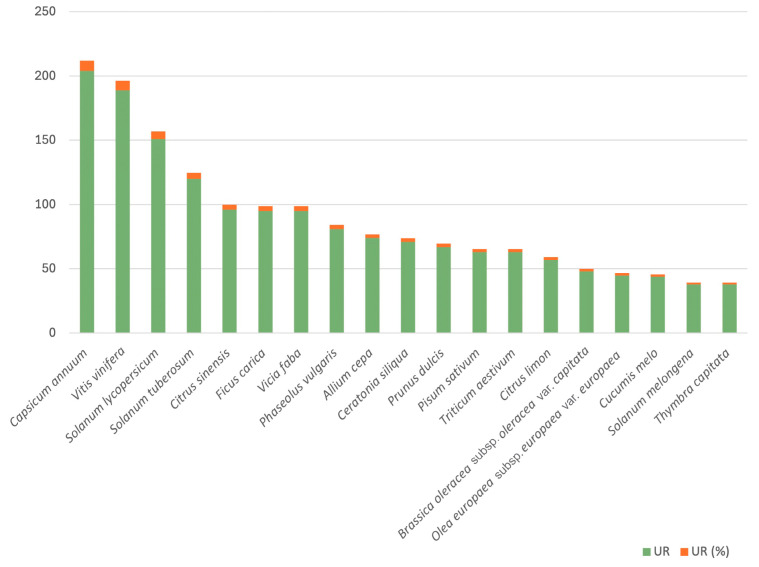
The most cited species with food uses.

The most cited species (Figure 5) is *Capsicum annuum* L. (204 URs, 7.91%). This species comprises numerous varieties cultivated in the majority of Ibizan home gardens. Renowned as an emblematic element of Ibizan cuisine, it features prominently in a multitude of traditional products and dishes (such as “coca”—a bread basis topped with vegetables—and the most famous of the island’s sausages, “sobrassada”, where it plays a predominant role for both colour and taste), lending its essence to various local names (e.g., “citró de matances”, highlighting its relevant role in pig slaughter, “matança del porc” in Catalan). The fruit of this plant is often directly consumed (fresh, fried in oil or cooked in the oven) and, also frequently (powdered or entire), serves as a condiment.

The second most cited species is *Vitis vinifera* (189 URs, 7.37%), which, like the previous one, also includes a large number of varieties. During the present research, we realised that many vine varieties and landraces in Ibiza (and the nearby smaller island Formentera) had not been studied in depth. Then, from this ethnobotanical prospection, complemented by one centred in traditional home gardens (González et al., in prep.), we characterised plant varieties from different viewpoints (ethnobotany, ampelography, genome size assessment, and genetic typification using microsatellites), and we could detect numerous cases of synonymy and similar phenomena among varieties and landraces and establish the characterisation of several new races [87,88]. Although the fruit of this species (grape) can be consumed dry (raisin), its most frequent uses are to be eaten fresh, as fruit, or to be used to make wine and all other related products, such as vinegar.

*Solanum lycopersicum* (151 URs, 5.88%) is also a species with several races but already a little distinct from the previous ones in terms of its uses, and the same applies to *Solanum tuberosum* (120 URs, 4.67%). Some of the varieties of tomatoes (“tomates de penjar”) are used to dip bread (a typical dish of the Catalan linguistic area) to make a base for other dishes or to eat fresh (salad). Potatoes are a typical crop in Ibizan home gardens. These tubers are usually consumed boiled in water, fried or baked. In addition to being used as food, potatoes are also widely used in Ibiza during the preparation of olives—not for eating but for checking the concentration of salt. When the potato floats after being added to the water with salt during preparation, the salt concentration is correct.

The flowers, leaves, fruit and fruit juices of *Citrus sinensis* (96 URs, 3.74%) have several food uses. The juice and fruit are often eaten fresh, but they are also one of the ingredients of “herbes eivissenques” (a herbal liqueur made with firewater).

Families and their URs are in Table S3 of the Supplementary Materials. The families with the most use reports (URs) are *Solanaceae* (519 URs, 20.20%), *Fabaceae* (384 URs, 14.94%), *Vitaceae* (189 URs, 7.35%), *Rosaceae* (174 URs, 6.77%), and *Rutaceae* (169 URs, 6.58%). Some of the families correspond to the most cited taxa that we mentioned above. The *Fabaceae* family appear among the most cited families because they include many species grown as food, such as the chickpea (*Cicer arietinum* L.), lentil (*Lens culinaris* Medic.), bean (*Phaseolus vulgaris* L.), pea (*Pisum sativum* L.), broad bean (*Vicia faba* L.) or chickling pea (*Lathyrus sativus* L.), but also other abundant species in the Balearic Islands, such as carob (*Ceratonia siliqua*), intended for both human and animal food, and alfalfa (*Medicago sativa* L.), a species largely used as fodder. Regarding the *Rosaceae* family, it should be noted that it includes many of the fruit trees often planted in home gardens, such as different species of the genus *Prunus* (*P. armeniaca* L., *P. avium* L., *P. domestica* L. and *P. dulcis* (Mill) Weeb.), of genus *Pyrus* (*Pyrus communis* L. and *P. malus* L. subsp. *mitis* (Wallr.) O.Bolòs et J.Vigo) and other edible fruit species, such as *Fragaria* × *ananassa* Duchesne, *Cydonia oblonga* Mill., *Sorbus domestica* L., *Mespilus germanica* L. and *Eriobotrya japonica*.

**Figure 6:**
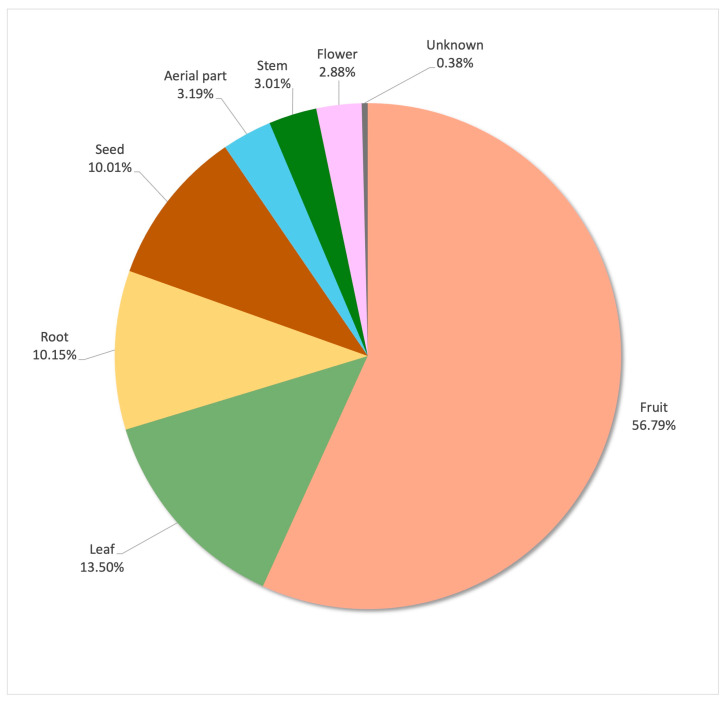
Parts of plant used as food.

**Figure 7:**
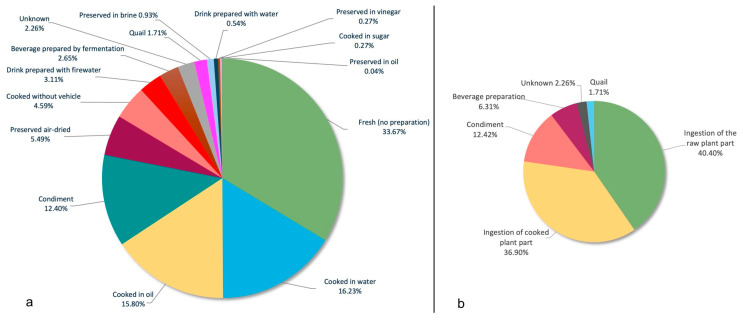
Preparation (**a**) and ingestion (**b**) of the food plants.

The parts of the plant used, grouped by category, can be found in Figure 6. The most used part of the plant is the fruit (1459 URs), which includes all the parts of the fruit and also the fructifications and infructescences. The leaves (347 URs) and the seeds (257 URs) occupy the second and third positions, respectively. Most of the parts of the plants are eaten fresh, with no preparation (865 URs), although ingestion is mostly raw (40.40%) or cooked (36.90%) (Figure 7).

### 2.5. Medicinal Plants and Food Plant Mixtures

Apart from the individual plant uses, this study inventoried 40 medicinal and 44 food mixtures, with 318 and 481 use reports (URs), respectively.

A total of 35 species are reported for the medicinal mixtures. The most cited species are *Rosmarinus officinalis* (48 URs, 15.09%), *Pinus halepensis* Mill. (34 URs, 10.69%), *Thymbra capitata* (L.) Cav. (31 URs, 9.75%), *Santolina chamaecyparissus* (26 URs, 8.18%) and *Lippia triphylla* (25 URs, 7.86%). The most frequent mixture is the “aigua de tot bosc” (water of the whole forest) and “herba de tot bosc” (grass of the whole forest) with 155 URs (48.74%), followed by the digestive and anti-inflammatory infusions and the “herbes eivissenques” (drink prepared with firewater), which is both a medicinal and alimentary mixture. Most species mentioned above are part of the “water of the whole forest”, which is a preparation with a plethora of medicinal uses (anticatarrhal, digestive, for trauma, etc.), including some veterinary remedies.

The parts of the plant used are the stem (45.6%), leaves (18.87%) and fruits (18.24%). The medicinal mixtures are mainly used to treat digestive diseases (25.47%), disorders of the skin and subcutaneous tissues (25.16%) and respiratory disorders (23.27%).

**Figure 8:**
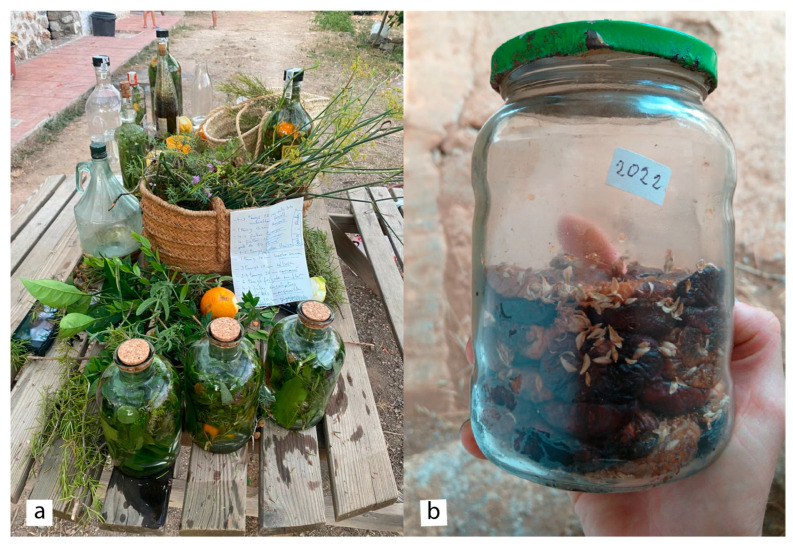
Process of making Ibizan herbs (**a**). Dried figs with leaves of *Thymbra capitata* (**b**).

Concerning the food mixtures, 69 taxa (66 species) in 44 mixtures (481 URs) have been cited, being *Citrus limon* (L.) Burm. (39 URs, 8.11%)*, Thymbra capitata* (33 URs, 6.86%), *Foeniculum vulgare* Mill. subsp. *piperitum* (26 URs, 5.41%)*, Olea europaea* L. subsp. *europaea* var. *europaea* (24 URs, 4.99%)*, Lippia triphylla* (19 URs, 3.95%)*,* and *Triticum aestivum* L. (19 URs, 3.95%), the species with more use reports. The parts of the plant most cited are the fruits (28.27%), stem (24.95%) and leaves (23.28%). The main products (typical alcoholic drink, in one case, or dishes—including desserts—of the traditional Ibizan cuisine, in the other cases) with their uses are “herbes eivissenques” (Ibizan herbs, a liqueur, 136 URs, 28.27%), “olives en salmorra” (olives preserved in brine, 45 URs, 9.36%), “cuinat” (cooked, 42 URs, 8.73%), “salsa de Nadal” (Christmas’s sauce, 22 URs, 4.57%), “plat de llegum” (pulses dish, 18 URs, 3.74%) and “macarrons de Sant Joan” (Saint John’s macaroni, 15 URs, 3.12%). First, it is remarkable that more than one-quarter of the reports of food mixtures corresponds to the liqueur “herbes eivissenques” (Figure 8), with the number of URs three times bigger than that of the following food mixture in the ranking. This is one of the most preserved ethnobotanical practices in Ibiza: even though the herbes have passed to the industrial and commercial levels—indeed, in concurrence with the liqueur with the same name on the island of Mallorca [28]—its traditional preparation remains as a quotidian current use, as we collected in the present paper and other authors have reported [8,54]. It is to be noted that medicinal properties (as a digestive, among others) are attributed to this herb’s mixture, placing it in the domain of functional foods [89]. Secondly, it is worth mentioning the second and third places in the food mixtures, which are very close to each other, each representing around 10% of the URs. These are preserved olives—one of the most emblematic Mediterranean products—and an alimentary product that has the simple name of “cuinat”, just meaning cooked, probably alluding to its basic character as a traditional island dish.

The index of taxon usefulness in mixtures (ITUM), the quotient of the number of reports of one taxon in mixtures and its total citations, whether simple or complex presentation, was calculated for the most cited taxa in the medicinal mixtures. The obtained values are the following: *Rosmarinus officinalis* (0.33), *Pinus halepensis* (0.32), *Thymbra capitata* (0.22), *Santolina chamaecyparissus* (0.20) and *Lippia triphylla* (0.19). In the same way, the ITUM has been calculated for the most cited taxa used in the food mixtures with the following results: *Citrus limon* (0.19), *Thymbra capitata* (0.23), *Foeniculum vulgare* subsp. *piperitum* (0.36), *Olea europaea* subsp. *europaea* var. *europaea* (0.12), *Lippia triphylla* (0.14) and *Triticum aestivum* (0.12).

### 2.6. Other Uses (Neither Medicinal nor Food)

Non-medicinal and non-food uses are included in the category “other uses”. In this study, 110 plant species (107 taxa including 12 infraspecific categories) were cited, comprising 72 wild and 38 cultivated plants belonging to 91 genera and 45 families, with a total of 505 use reports for this category. The most cited families were *Poaceae* (79 URs, 15.58%), *Pinaceae* (42 URs, 8.28%), *Lamiaceae* (33 URs, 6.51%), *Rutaceae* (33 URs, 6.51%), *Cistaceae* (30 URs, 5.92%) and *Cupressaceae* (30 URs, 5.92%).

The most used parts of the plants in this category are the stem (133 URs, 26.23%), leaves (65 URs, 12.82%), stem with leaves and/or branches (46 URs, 9.07%), aerial parts of the plant (44 URs, 8.68%) and plants living in situ (29 URs, 5.72%).

The 20 most cited plants are shown in Table 4. *Pinus halepensis* was the most cited taxa by the informants (42 URs, 8.28%), mostly used to build toys. Also, the resin obtained is used to produce a traditional adhesive known as “pega”, which has been historically used to waterproof and insulate the soles of espadrilles. *Lygeum spartum* L. was reported 32 times (6.31%). The leaves were the most frequently cited part, as they were—and still are—used to craft strings and cords, some of which are used in the making of espadrilles. *Cistus albidus* L., accounting for 5.72% (29 URs) of the citations, was mostly used because of its stem with leaves and or branches, as well as its leaves. It is usually used as a dish sponge or toilet paper.

The other uses the importance index (the quotient of the total other non-medicinal and non-food use reports and the number of taxa with other uses), an adaptation of the other uses category of the medicinal importance index [25], is 4.71, much lower than those calculated for medicinal and food uses. The informant consensus factor for food uses is 0.79, which is not very low in absolute value but is essentially the lowest value for this parameter in general and for medicinal and food uses in this work. These low values probably suggest an erosion of knowledge.

Even if this section is the one with the most subcategories, the reports of use are scarce, indicating a strong erosion suffered by this traditional knowledge, partly due to changes in the habits and way of life of the Ibizan and world population and partly because many tasks and many trades related to rural life have disappeared. The transition from manual jobs to new technologies and globalisation are factors that have influenced this impoverishment. The lack of vertical transfer of knowledge due to the change in customs and family relationships may also have played an important role.

### 2.7. Vernacular Names

To name the 254 taxa constituting the general ethnofloristic catalogue of Ibiza island, the informants have reported 674 names (3244 URs), 602 (89.32%) of which in Catalan, 70 (10.39%) in Spanish, and 2 (0.30%) in English.

As for quantitative ethnobotany data linked to names, the ethnophytonymy index for wild plants is 12.4, indicating the percentage of taxa with folk Catalan names present in the flora and coinciding with the index of ethnobotanicity since, in this study, all plants with registered uses also have at least one vernacular name. Finally, the linguistic diversity index in phytonymy is 2.61, indicating a linguistic richness of the territory since there are, as a mean, ca. three names for one plant. This figure is among the high ones in the Catalan linguistic domain [3] and is very similar to the one obtained in the neighbouring Mallorca [26].

According to the relevance of agronomic ethnoflora in this study, the names of landraces of cultivated plants account for a non-negligible part of the phytonymic diversity. It is worth mentioning that we collected 272 names of landraces for 43 taxa (42 species) belonging to 33 genera and 14 families. As it can be easily observed, a significant part of the phytonyms collected (40.24%) is formed by the landrace’s names. As an example, the two plants with more landrace names, *Capsicum annuum* and *Vitis vinifera*, have 26 and 52 names for 8 and 19 races, respectively. This indicates richness in both local germplasm and biocultural-associated knowledge.

On the opposite side of the plants with many names, we find some taxa for which very few or just one name is reported. This is the case, for instance, of “guixa”, the only name for *Lathyrus sativus*, and *Thymbra capitata*, only known as “frígola”. This can be interpreted as cases of scarce dialectological variability.

Folk plant names very often serve as indicators of certain characteristics of the plants they designate. We can add a few more to the above-commented phytonym “citró de matances” (see food plant subsection). Some names may bring information on plant use, such as “herba-sana” (healthy herb, *Mentha spicata* L.), “herba de formatjar” (cheese-making herb, *Cynara cardunculus* L.), and “raspall” (“brush”, *Cneorum tricoccon* L., used to make brooms). This indicates a very solid and long tradition of using certain plants, which is reflected in their names. In other cases, morphological (“pebrera vermella”, red pepper, *Capsicum annuum*), ecological (“fonoll marí”, marine fennel, *Crithmum maritimum* L.) or other aspects, such as similitude with other plants (“bleda borda”, bastard beet, *Beta vulgaris* L. subsp. *maritima* (L.) Arcang.) are reflected in the phytonyms. In addition, some plant names are useful to characterise the Ibizan subdialect of Catalan language. It belongs to the eastern set of dialects, but the use of the name “dacsa” (*Zea mays* L.) shows its linkages with the western set, particularly with the Valencian subdialect. In Mallorca and Menorca [22,26], the names usually employed to designate this taxon are “blat de les Índies” or “blat dindi” (in agreement with other eastern subdialects, such as northern one), whereas “dacsa” is limited, in eastern dialects, to Ibiza and also to Formentera [60] islands, the closest to the Valencian territory. All this shows one more time the robustness and relevance of the biocultural pool generated by the interaction between humans and plants, clear at both tangible (uses) and intangible (names and some practices) levels.

## 3. Materials and Methods

### 3.1. Study Area

The territory investigated is Ibiza island, which has an extension of 571.79 km^2^ and has 154,210 inhabitants. It encompasses five municipalities: Eivissa city (50,715 inhabitants, with an extension of 1.11 km^2^), Santa Eulària des Riu (40,458, 15.351 km^2^), Sant Josep de sa Talaia (28,813, 15.885 km^2^), Sant Antoni de Portmany (27,431, 12.670 km^2^), and Sant Joan de Labritja (6703, 12.153 km^2^) [14].

Ibiza was formed during the tertiary era, as a result of Alpine orogenesis, at the end of the Oligocene and the beginning of the Miocene. Like the whole Balearic Islands, it is a prolongation of the Baetic mountains, although nowadays, it has very low mountains, not reaching much more than 400 m a.s.l., with the highest altitude of the island Sa Talaiassa at 475 m [90]. There is no permanent watercourse on the island, only seasonal streams. Due to the calcareous nature of geological materials, soils are basic, with a high content of calcium.

The Ibizan climate is typically Mediterranean, with dry summers. The average annual temperature is 17.5 °C, with a moderate annual thermal oscillation or amplitude of about 15 °C, ranging from 11 °C in January to 26 °C in August. The summer is long and warm—five months with average temperatures of around 18 °C or more—and the winters are very mild. In reality, there is no true winter since, meteorologically speaking, the average temperature in the coldest month, January, does not go below 10 °C, and in fact, days with temperatures below 0 °C do not usually occur.

The floristic catalogue of native species of Ibiza was estimated by Rita and Payeras [91] to include 921 infraspecific taxa, 44 of which are endemic, belonging to 879 species classified within 99 families. These figures comprise only autochthonous plants. Considering that the Balearic Islands have a proportion of non-native species of 56% [92], if Ibiza has the same proportion of these species, the approximate number of non-native species corresponding to the largest of the Pityusic Islands would be 170, and then, the wild flora of Ibiza could be estimated at around 1049 species. Including the difference between infraspecific taxa and species (42), the total rises to approximately 1091 taxa. This figure is used to calculate the ethnobotanical index, acknowledging the potential for a higher proportion of non-native species.

The island is covered by Mediterranean vegetation [93,94,95,96,97], primarily characterised by loosely structured *Pinus halepensis* forests, allowing for a shrub layer composed of other gymnosperms such as *Juniperus phoenicea* L. and *J. oxycedrus* L., along with other species. In addition, there is rich coastal vegetation on both cliffs and sandy dunes and beaches. On cliffs, *Limonium* species and *Crithmum maritimum* are particularly relevant, as well as *Asperula cynanchica* L. subsp. *paui*—endemic of the Pityusic area and a small part of the Iberian coast closest to these islands—found at higher altitudes, and *Cneorum tricoccon* and *Hippocrepis balearica* jacq. In sandy dunes and beaches, typical species include *Eryngium maritimum* L., *Euphorbia paralias* L., *Elymus farctus* (Viv.) Runemark and *Pancratium maritimum* L. In coastal areas, salt marshes, where there are salt flats and halophytic vegetation, with *Atriplex portulacoides* L. and other *Amaranthaceae* of genera *Salicornia* and *Sarcocornia*, are also present.

### 3.2. Data Collection

The information was gathered through semi-structured interviews [98] conducted individually or in groups with informants between the years 2005 and 2023. Participant observation [98] was commonly performed, since the first author was born in Ibiza, resides there, and, with a background in agronomic and environmental sciences, is very often in contact with rural Ibizan society. Typically, these informants are of advanced age and possess intimate knowledge of their surroundings, particularly regarding plants and their uses. Informants were selected using the snowball sampling method [99] and previously informed about the objective of the interview. Verbal consent was always obtained instead of written consent, as we considered that the latter could be more challenging to implement, potentially introducing unnecessary bureaucracy, while always adhering to the principles of the International Society of Ethnobiology [100]. Most interviews were developed in the Catalan language, and very few in the Spanish language, in both cases without the need for an interpreter. All the information was transcribed and entered into our research group’s internal database (http://gestio.etnobotanica.cat/index.php, accessed on 22 September 2024).

For taxon identification and nomenclature, the *Flora Manual dels Països Catalans* [101] has been used, with *Plants of the World Online* [102,103] for the plants (cultivated or commercial, for instance) not present in the former work, only devoted to wild flora. The most recent Angiosperm Phylogeny Group’s structuring [103] was followed for assigning botanical families. A herbarium voucher for each mentioned taxon was deposited at the BCN Herbarium of the Centre de Documentació de Biodiversitat Vegetal, Universitat de Barcelona.

All data were analysed using Microsoft Excel [104], with use reports (URs) as the unit of analysis. It is important to note that a use report refers to the citation of a taxon or a part thereof for a specific use by an informant.

To assess the state of ethnobotanical knowledge in the studied area using quantitative ethnobotany, various indices were calculated. Among them is the ethnobotanicity index (EI) [77], which is the quotient of the number of plants with uses (only taking into account the native plants) and the total number of plants of the flora of the studied area, expressed as a percentage. The informant consensus factor (F_IC_) [80] has also been calculated, which is the ratio of the number of URs minus the number of used taxa to the number of URs minus one; it indicates a more robust and reliable information corpus when closer to 1. The relative frequency of citation (RFC) is obtained by dividing the number of informants who mentioned the use of the species by the total number of informants [105]. The quotient of the total use reports for a specific use category and the number of taxa possessing this use [25] was also calculated. This index was initially described for medicinal uses and called the index of medicinal importance (MI), but it can be applied to other uses, such as food. Regarding the vernacular names, the ethnophytonymy index [106] was calculated as a percentage of taxa with Catalan names present in the flora. For plant names, one more index has been considered: the linguistic diversity index in phytonymy [107], which is the mean number of folk names for the cited plants, evaluating the linguistic richness of a territory independently of its flora. For preparations based on more than one plant taxon, the index of taxon usefulness in mixtures (ITUM) [108], which is the quotient of the number of reports of one taxon in mixtures and its total citations, whether simple or complex presentation, has also been calculated. The value of this index ranges from 0 to 1, approaching zero when the number of citations in simple presentation is higher than the number of citations in mixtures, and it will be one when the taxon is used only in mixtures.

## 4. Conclusions

Ethnobotanical research helps preserve local knowledge and serves as a database for the future. This study has allowed the creation of an ethnobotanical catalogue, which Ibiza island did not have until now.

The results of this study show that although knowledge about other plant uses apart from medicinal and food ones—such as making cords or tools—has suffered a significant decline on the island due to the shift from a rural lifestyle to a tourism-based economy, biocultural relevance and plant knowledge still exist on the island, which can be seen from the number of taxa cited in this study, their numerous and diverse uses and the robust ethnobotanical indexes.

Further research should be conducted comparing similar territories with a strong tourism influence to determine whether the results found here are repeated.

## Figures and Tables

**Table 1:** Interviews and informants in each municipality.

Municipality	Number of Interviews (%)	Number of Informants *	Male (%)	Female (%)
Eivissa	6 (6.32)	10	60	40
Santa Eulària des Riu	26 (27.37)	21	76.19	23.81
Sant Antoni de Portmany	18 (18.95)	20	45	55
Sant Joan de Labritja	10 (10.53)	14	28.57	71.43
Sant Josep de sa Talaia	35 (27.37)	36	69.44	30.56
Total	95	101		

* Some of the informants have been interviewed more than once.

**Table 2:** Pharmaceutical forms.

Pharmaceutical form	UR	%
Infusion	221	45.76
Without pharmaceutical form (direct use)	105	21.74
Bathroom	22	4.55
Unknown to the informant/Not recorded	19	3.93
Mouthwash	18	3.73
Extract	16	3.31
Medicinal wine/vinegar	11	2.28
Prepared with honey	10	2.07
Aerosol	9	1.86
Decoction	8	1.66
Macerated in oil	8	1.66
Plaster	8	1.66
Gargling	6	1.24
Poultice	6	1.24
Injectable	4	0.83
Liniment	4	0.83
Alcohol tincture	3	0.62
Sugary fluid, watery juice	3	0.62
Medicinal species	2	0.41

**Table 3:** The top 20 most cited species with food uses.

Species	UR	UR (%)
*Capsicum annuum* L.	204	7.941
*Vitis vinifera* L.	189	7.357
*Solanum lycopersicum* L.	151	5.878
*Solanum tuberosum* L.	120	4.671
*Citrus sinensis* (L.) Osbeck	96	3.737
*Ficus carica* L.	95	3.698
*Vicia faba* L.	95	3.698
*Phaseolus vulgaris* L.	81	3.153
*Allium cepa* L.	74	2.880
*Ceratonia siliqua* L.	71	2.764
*Prunus dulcis* (Mill.) Weeb.	67	2.608
*Pisum sativum* L.	63	2.452
*Triticum aestivum* L.	63	2.452
*Citrus limon* (L.) Burm.	57	2.219
*Brassica oleracea* L. subsp. *oleracea* var. *capitata* L. *f. capitata*	48	1.868
*Olea europaea* L. subsp. *europaea* var. *europaea*	45	1.752
*Cucumis melo* L.	44	1.713
*Solanum melongena* L.	38	1.479
*Thymbra capitata* (L.) Cav.	38	1.479
*Ipomoea batatas* Poir.	35	1.362

**Table 4:** The 20 most cited plant taxa with other uses.

Species	UR	UR (%)
*Pinus halepensis* Mill.	42	0.083
*Lygeum spartum* L.	32	0.063
*Cistus albidus* L.	29	0.057
*Arundo donax* L.	25	0.049
*Cneorum tricoccon* L.	20	0.039
*Prunus dulcis* (Mill.) Weeb.	17	0.034
*Juniperus oxycedrus* L. subsp. *oxycedrus*	16	0.032
*Agave americana* L.	14	0.028
*Juniperus phoenicea* L. subsp. *turbinata* (Guss) Parl.	14	0.028
*Nicotiana rustica* L.	14	0.028
*Ocimum basilicum* L.	12	0.024
*Scirpus holoschoenus* L.	11	0.022
*Ceratonia siliqua* L.	10	0.020
*Olea europaea* L. subsp. *europaea* var. *europaea*	10	0.020
*Teucrium polium* L.	10	0.020
*Vitis vinifera* L.	9	0.018
*Opuntia maxima* Mill.	7	0.014
*Cannabis sativa* L.	6	0.012
*Ferula communis* L. subsp. *catalaunica* (Pau) Sánchez-Cux and M. Bernal	6	0.012
*Foeniculum vulgare* Mill. subsp. *piperitum* (Ucria) Cout.	6	0.012

## Data Availability

Data related to this article are available in the Supplementary Materials; any other information can be consulted to the authors.

## Supplementary Materials

The following supporting information can be downloaded at https://www.mdpi.com/article/10.3390/plants14060890/s1, Table S1: Med use; Table S2: Food use; Table S3: Food botanical families; Table S4: Other use; Table S5: Toxic use; Table S6: Med mix UR; Table S7: Food mixed; Table S8: Tax_inf_fam; Table S9: Names.
